# Perceptions of Students in Different Phases of Medical Education of Educational Environment: Ankara University Faculty of Medicine

**DOI:** 10.3885/meo.2008.Res00267

**Published:** 2008-06-09

**Authors:** M. Demirören, Ö. Palaoglu, S. Kemahli, F. Özyurda, I.H. Ayhan

**Affiliations:** Faculty of Medicine, Department of Medical Education and Informatics, Ankara University, Ankara, Turkey

**Keywords:** educational environment, DREEM, medical education, PBL

## Abstract

The present study was undertaken to identify the perceptions of students about their educational environment in a newly restructured curriculum. The Turkish version of the DREEM questionnaire (total score: 200) was used to diagnose the strengths and weaknesses of the curriculum which is known to be a major determinant of educational environment. Five hundred fifty three students (years 1, 3, 5) voluntarily replied to the questionnaire. The mean DREEM score was found to be 117.63 (58.8%). The mean scores for the whole DREEM questionnaire and the five essential domains were found to be significantly different in different phases of medical education. The scores were found to be highest (123.65) for year 3 students and lowest (109.39) for year 5 students. The results are the first data of a curriculum reform obtained from the students about the educational environment and give important feedback to curriculum planners and change managers of the faculty for necessary improvement.

A traditional undergraduate curriculum had been conducted at Ankara University Faculty of Medicine (AUFM) since its founding in 1945. Starting with students admitted in 2002, the traditional, discipline-based curriculum has been transformed to an innovative curriculum stemming from the principles of the Edinburgh Declaration, i.e. student-centered, integrated, problem-based, competency-based and community-based principles of contemporary medical education. After implementing the new curriculum, the curriculum planners decided to assess the perceptions of students about the new educational environment, taking into account the very well known end point concept that any curriculum change is actually and essentially an environmental change.^1^,^2^

The present study was carried out to identify the perceptions of students undertaking the restructured curriculum in order to find:

1. How students perceive the new educational environment
2. Whether there is any difference between the perceptions of students according to the stages of medical education, i.e., the preclinical and clinical stages
3. What are the perceived strengths and weaknesses of the new curriculum?

## Methods

The Dundee Ready Educational Environment Measure (DREEM) questionnaire, a generic, highly reliable and diagnostic inventory, was used as a measure of students’ perceptions about the educational environment in AUFM. It was developed for undergraduate health professionals. It has been shown to be independent of culture, and its translated version to various languages has been used in many countries^3^. It is a 50-item inventory where each item is scored using a five-point Likert scale with 0 = strongly disagree, 1 = disagree, 2 = unsure, 3 = agree and 4 = strongly agree. Seven negative items are scored in reverse order. The 50 items have been categorized into five domains whose scores are as follows:

**Table:** 

		Max
	Items	Score
Student's Perceptions of Learning	12	48
Student's Perceptions of Teaching	11	44
Student's Academic Self-perceptions	8	32
Student's Perceptions of Atmosphere	12	48
Student's Social Self-perceptions	7	28

Total possible score 200

The questionnaire was translated into Turkish by the faculty members of Department of Medical Education and Informatics and reviewed by the Assessment and Evaluation Department of Ankara University School of Educational Sciences. The translated inventory was pretested within a group of 24 students for their suggestions about the clarity. The finalized version is identified as the Turkish Version of DREEM.

The DREEM questionnaire was given to Year 1, Year 3 (at the end of preclinical phase) and Year 5 students (at the end of clinical phase just before rotating). A total of 668 students (Year 1 *n*=285, Year 3 *n*=245, Year 5 *n*=138) were asked to answer the Turkish Version of DREEM. The questionnaire was distributed to the students on the week of registration for second term (January, 2007) following a brief explanation of the objectives and data processing procedures, including anonymity and the importance of voluntary-based participation.

The resulting scores for domains were interpreted using the guide proposed by McAleer and Roff.4 For statistical analysis of the data for the whole 50 item inventory, scores for categorized domains and each item were both expressed as Mean±Standard Deviation (SD) and percent value. Data were analyzed using the statistical package SPSS. One-Way ANOVA and independent samples *t*-test were used to identify the significance between subgroups.

## Results

The students’ reply rate was 82.8% (*n*=553). This rate was 89.5% (*n*=255) for Year 1, 78.4% (*n*=192) Year 3 and 76.8% (*n*=106) for Year 5 students.

The Turkish Version of DREEM mean score for all the students replying was 117.63±21.655 (58.8%). By year, the mean score was 116.53±20.940 (58.3%) for students in Year 1, 123.65±20.870 (61.8%) for Year 3 and 109.39±21.785 (54.7%) for Year 5. These mean scores were determined to be statistically significantly different from each other [F(2-550) = 16.290, *p*<.01].

Mean±SD scores for the five essential domains of the DREEM questionnaire for the three groups of students are shown in Table 1. When the total mean domain scores were compared between the groups, scores of year 5 students for “Student's Perception of Learning” (25.79) and “Student's Perception of Teaching” (24.96) were significantly lower than year those of years 1 and 3. Mean cores of year 3 students for “Student's Academic Self-perception” (19.99), “Student's Perception of Atmosphere” (=28.51) and “Student's Social Self-perception” (17.38) were significantly higher than those domain scores for years 1 and 5 students.

**Table 1:** Mean Score ± SD of three students groups for the five essential domains of DREEM

Domains of DREEM	Max score	Mean Score ± SD				p
		Year 1	Year 3	Year 5	All the groups
		(n = 255)	(n = 192)	(n = 106)	(n = 553)
Students' Perception of Learning (SPL)	48	27.75±6.560	29.03±6.459	25.79±6.238	27.82±6.553	*
Students' Perception of Teachers (SPT)	44	27.64±4.937	28.74±5.092	24.96±5.825	27.51±5.332	*
Students' Academic Self-perception (SAP)	32	17.99±4.179	19.99±3.754	18.50±4.395	18.78±4.171	*
Students' Perception of Atmosphere (SPA)	48	26.66±6.239	28.51±6.348	25.23±6.457	27.03±6.422	*
Students' Social Self-perception (SSP)	28	16.49±3.375	17.38±3.600	14.92±3.732	16.50±3.622	*
Total DREEM score	200	116.53±20.940	123.65±20.870	109.39±21.785	117.63±21.655	*

* represents p < 0.05

The domain scores for the whole group were compared on a percentage basis because of the different maximum score of each domain. The highest percent score was observed for the “Student's Perception of Teachers” domain (62.5%) and the lowest for the “Student's Perception of Atmosphere” (56.3%).

The scores were compared on the basis of the items as well. Of the 50 mean item scores, nine were found to be below 2.0, two to be above 3.0 for all the students, as shown in Table 2. The lowest being 1.45 (Item 27: I am able to memorize all I need) and the highest being 3.26 (Item 2: The teachers are knowledgeable).

**Table 2:** DREEM items with scores greater than 3.0 or less than 2.0 (*n* = 553)

Item		Mean score*
2	The teachers are knowledgeable	3.26 (SPL)
15	I have good friends in this school	3.18 (SSP)
3	There is a good support system for students who get stressed	1.70 (SSP)
4	I am too tired to enjoy this course	1.99 (SSP)
9	The teachers are authoritarian	1.65 (SPT)
14	I am rarely bored on this course	1.79 (SSP)
17	Cheating is a problem in this school	1.76 (SPA)
24	The teaching time is put to good use	1.53 (SPL)
25	The teaching over-emphasizes factual learning	1.89 (SPL)
26	Last year's work has been a good preparation for this year's work	1.97 (SAP)
27	I am able to memorize all I need	1.45 (SAP)

*SPL: Students' Perception of Learning; SPT: Students' Perception of Teachers; SAP: Students' Academic Self-Perception; SPA: Students' Perception of Atmosphere; SSP: Students' Social Self-Perception

On comparing the mean item scores for the three groups of students, seen in Table 3 (Appendix), only seven items (Item No: 13, 27, 28, 30, 35, 46, 50) were found to show no significant difference between the groups.

## Discussion

Teaching is known to be not only related to giving information and sharing experiences but producing a contextually or/and environmentally related learning as well.^5^ A medical school is an environment in which students are expected to experience various learning activities. It is very well known that curriculum is the most important determinant of the learning environment and learning environment is the most important determinant of the behavior of all the parties of education. Thus, it is expected that any curriculum change should involve changes in educational environment, management and organization to result in the predicted behavioral changes.^1^,^2^

The curriculum change was expected to build up a better educational environment, perceived as good by students. A continuous improvement in the educational environment of a curriculum is only possible by defining its weaknesses and strengths, thus, monitoring the perceptions of students at different stages about the educational environment is critical. To that end, the DREEM, a questionnaire used to assess the educational climate of health professional/medical schools, was used to identify how students perceived their educational environment.

The overall reply rate of 82.8% is an acceptable rate for voluntary-based participation. The DREEM mean score of our students (117.63/200) is higher than the scores found by Till (78-113/200), Al-Halima et al. (102/200, Jiffry et al. (108/200) and Bassaw et al. (103-113/200).^4^,^6^–^8^ On the other hand, while the scores of our students are similar to those of Nigerian students (118/200), the scores found by Roff et al. for Nepalese students (130/200) and by Miles and Leinster (143/200) for British students are higher than our scores.^9^,^10^

The results of the present study show that Year 3 students have a more positive perception of the educational environment than year 1 and 5 students. Although Till has found the lowest DREEM score for year 3 students^6^, our results are comparable with the results of Jiffry et al. and Roff et al. from Nepalese students showing the highest scores for year 3 students.^4^,^9^ The recent results indicate that students show a progressive increment of their mean scores about the educational environment until the clinical stages of medical education. This increment may be the result of the early exposure to patients in the new curriculum in our instance. Moreover, an inevitable adaptation period for year 1 students, just graduated from 11 years of a traditional education system to a completely different learning and teaching environment, should not be disregarded as a factor for the lower scores of year 1 students as well. On the other hand, it is critical to discuss why the scores of year 5 students are the lowest scores. At a first glance, it can be argued that this group of students is the first group to experience the newly restructured clinical stage of education and should be accepted as an opportunity to find out the weaknesses of the curriculum and environment.

To better define the weaknesses and strengths, the five essential domains and corresponding items of DREEM were comparatively interpreted. When the guide of Mc Aleer and Roff was used to interpret the mean scores, all students perceive “*a more positive approach*” (27.82/48) for their learning; “*moving in the right direction*”(27.51/44) for their teachers; “*feeling more on the positive side*” (18.78/32) for their academic self-perception; “*a more positive atmosphere*” (27.03/48) for the atmosphere and “*not too bad*” (16.50/28) for their social self-perception.4 These results should be stimulating for the curriculum planners to transform students’ perceptions about their educational environment to a higher level.

**Students’ Perception of Learning (SPL)** - The agreement of all student groups on Item 13 (The teaching is often student-centered) is critically important regarding the main goal of the curriculum change, its being student-centered. The scores of Items 24 (The teaching time is put to good use) and 25 (The teaching over-emphasizes factual learning) found below 2.0 have to be considered critically as well. Their perception of an over-emphasis on factual learning can be discussed in the context of the assessment methods used because it is very well known that assessment has the ability to drive learning.^11^

Perceptions of Year 5 students that education is more teacher-centered (2.72), less motivating to the learner to be active (2.27), giving less importance to long-term learning (2.09) and being less sufficient to develop competency (2.15) are not as positive as both year 1 and 3 students. Such a significant differential perception might arise from the different learning environments in preclinical and clinical stages as has been emphasized previously elsewhere.^12^ It has been suggested that clinical education actually is inconsistent, unpredictable, immediate and devoid of continuity^13^ and that it is critical to structure clinical, especially bed-side, training.^14^ The present study's results indicate the need to recheck clinical training in the new curriculum.

**Students’ Perception of Teachers (SPT)** - On a percentage-based evaluation, Student's Perception of Teachers was found to be the highest of all the domains for the whole group. All the student groups scored Item 2 (The teachers are knowledgeable) over 3.0. On the other hand, Item 9 (The teachers are authoritarian) was scored below 2.0, which may indicate that teachers are still wearing their traditional hats.

Year 5 students have the most negative perception (24.96 of a maximum 44 points) about teachers. Their most negative perception is for giving feedback and making constructive criticism (Items 29 and 32 with scores of 1.56 and 1.74, respectively). These results are important for the clinical education period. Clerkships can be defined as educational black boxes; it has been shown that a positive learning atmosphere, fostering active student participation, taking responsibility, effective supervision and giving positive feedback is vital, making the role of clinical trainer critical for clinical training^15^,^16^. The above results indicate that there is a need for supporting trainers in terms of clinical training skills.^13^,^17^

**Students’ Academic Self-perception (SAP)** - While year 3 students achieved the highest score (19.99, maximum of 32 points) for academic self-perception, year 1 students achieved the lowest (17.99). Such a discrepancy might be related to the lesser experience of year 1 students in educational and assessment measures.

The lower scores of Year 5 students need considerable attention just before graduation. The progressive decrease of scores for Item 21 (I feel I am being well prepared for my profession) from Year 1 to Year 5 students may be discussed by the more positive approach of year 1 students to medical education, parallel to the suggestions of Hilton and Slotnick^18^ and the changing structure of clinical education where the traditional apprenticeship role plays less of a role.^12^

The significant difference between the results obtained for Item 41 (My problem-solving skills are being well developed here) needs to be discussed as well. The higher scores of year 3 students compared to year 1 students might point out the increased experience of students with PBL where problem solving skill is a generic outcome.^19^ Contrarily, the lower scores of year 5 students compared to year 3 students seem to be an unexpected result from the point of view that clinical education is based on solving real patient problems.

**Students’ Perception of Atmosphere (SPA)** - It is very well known that the atmosphere perceived actually represents the real-life educational environment and thus the dynamism of the curriculum.^2^ Year 3 students have a more positive perception (28.51, maximum 48 points) for the atmosphere than Year 1 (26.66) and Year 5 students (25.23). Such a result may be discussed with the discrepancy between the preclinical and the clinical educational environments. First of all, the clinical (hospital) environment is a real, authentic environment and is not suitable for effective learning unless well planned and organized.^20^ Although the AUFM clinical education has been restructured according to contemporary approaches and with attention to the common pitfalls of clinical education, the results clearly show that the actual practice needs a critical review.

**Students’ Social Self-perception** - All the students participating in the study share the perception that there is not a good supporting system for those who get stressed. Year 5 students have the most negative perception for Item 3 (There is a good support system for students who get stressed). This result should be discussed not only at the curriculum planners’ level but should also urge the administrators to establish a social and academic support service for students.

**Limitations** - These results are the first indicators of how students perceive the educational environment of the new curriculum. Thus they can be accepted as a baseline for this cohort. As there are no comparable data for the traditional curriculum, it is not possible to compare the effects of the new curriculum to the former curriculum on students’ perception of the educational environment. It is known that monitoring any change within the same cohort is possible with longitudinal studies. But it must be taken into consideration that in longitudinal studies students’ perception about learning environment in the preclinical and clinical phases of medical education may actually be different.^21^ Moreover, the present study offers no comparison with the ideal expectation of students of a medical school learning environment.

It is very well known that learning environment has a considerable effect on the approach of students to learning and their academic success.^1^,^6^ Thus, investigating the correlation between perception of learning environment and the academic success of students who participated in the study would be a good study to pursue.

One of the most important limitations of the study is the use of a questionnaire to assess the perception of learning environment because there is the possibility of leaving out some components of a specific context.^12^ The results of this study, therefore, should be further supported by new studies.

## Conclusion

The learning environment is not only an important determinant of curriculum but is also a striking index of the behavior of both students and trainers. Thus, the behaviors of medical students are determined not only by their personality but by the characteristics of the learning environment as well.^1^

Successful management of any change in education is only possible with systematic feedback and assessments. The DREEM has been useful in identifying the strengths and limitations of the new curriculum. Results mainly show that students perceive the environment as student-centered. However they are not happy with the time schedule, they indicate too much factual learning and inefficient social support, and they feel weary. The second inference of the study is that students share a more negative perception on the learning environment and trainers during the clinical phase. The effects of hidden curriculum might be important to explain the relatively lower scores of year 5 students for the main domains of DREEM. It has been shown that hidden curriculum is tightly associated with the social and the physical environment and may be more effective than the manifest curriculum.^22^ There are examples of the effects of hidden curriculum on loss of idealism, emotional neutralization, change of ethical integrity, the learning of less formal aspects of “good doctoring”. Thus, any change in the manifest curriculum should be paralleled with changes in the hidden curriculum as well;^23^ further studies of data about the hidden curriculum in our medical school are needed.

Consequently, the time schedule of the new curriculum, trainers’ behavior, clerkship training, the social/academic and psychological support system, and the constructivist approach in learning have come out to be the main intervention areas for the development of the new curriculum and, thus, the learning environment.

## Appendix

**Table 3:** Mean Scores for each DREEM item of the three groups of students

		Mean Scores (max.score 4)			
Domains/Item No/Items of DREEM Questionnaire		Year 1	Year 3	Year 5	
		(n = 255)	(n = 192)	(n = 106)	p<0.05
**Students’ Perception of Learning (SPL)**					
1	I am encouraged to participate in class	2.58	3.03	2.42	1,2
7	The teaching is often stimulating	2.17	2.48	1.93	1,2,3
13	The teaching is student-centered	2.83	2.80	2.66	**NS**
16	The teaching is sufficiently concerned to develop my competence	2.66	2.38	2.15	1,3
20	The teaching is well focused	2.56	2.57	2.20	2,3
22	The teaching is sufficiently concerned to develop my confidence	2.22	2.34	1.82	2,3
24	The teaching time is put to good use	1.34	1.89	1.34	1,2
25	The teaching over-emphasizes factual learning	1.72	1.91	2.25	1,2,3
38	I am clear about the learning objectives of the course	2.29	2.27	1.95	2,3
44	The teaching encourages me to be an active learner	2.43	2.49	2.27	3
47	Long-term learning is emphasized over short-term	2.46	2.35	2.09	2,3
48	The teaching is too teacher-centered	2.42	2.54	2.72	3
**Students’ Perception of Teachers (SPT)**					
2	The teachers are knowledgeable	3.08	3.50	3.26	1,2,3
6	The teachers are patient with patients	2.42	2.79	2.59	1,2,3
8	The teachers ridicule the students	2.70	2.71	1.85	2,3
9	The teachers are authoritarian	1.76	1.63	1.41	3
18	The teachers have good communications skills with patients.	2.60	2.77	2.31	1,2,3
29	The teachers are good at providing feedback to students	2.23	2.16	1.56	2,3
32	The teachers provide constructive criticism here	2.20	2.20	1.74	2,3
37	The teachers give clear examples	2.60	2.83	2.52	1,2
39	The teachers get angry in class	2.71	2.64	2.39	2,3
40	The teachers are well prepared for their class	2.43	2.64	2.47	1
50	The students irritate the teachers	2.92	2.88	2.86	**NS**
**Students’ Academic Self-Perception (SAP)**					
5	Learning strategies which worked for me before continue to work for me now	2.40	2.58	2.31	2
10	I am confident about my passing this year	2.82	3.14	2.94	1,2
21	I feel I am being well prepared for my profession	2.50	2.28	1.91	1,2,3
26	Last year's work has been a good preparation for this year's work	1.62	2.43	1.99	1,2,3
27	I am able to memorize all I need	1.47	1.50	1.33	**NS**
31	I have learned a lot about empathy in my profession	2.30	2.74	3.06	1,2,3
41	My problem-solving skills are being well developed here	2.38	2.60	2.31	1,2
45	Much of what I have to learn seems relevant to a career in medicine	2.50	2.73	2.65	1
**Students’ Perception of Atmosphere (SPA)**					
11	The atmosphere is relaxed during the ward teaching	2.33	2.38	2.10	2,3
12	This school is well time-tabled	1.97	2.22	1.68	1,2,3
17	Cheating is a problem in this school	1.59	1.91	1.91	1,3
23	The atmosphere is relaxed during the lectures	1.99	2.45	2.13	1,2
30	There are opportunities for me to develop inter-personal skills	2.43	2.43	2.27	**NS**
33	I feel comfortable in class socially	2.53	2.70	2.48	2
34	The atmosphere is relaxed during seminars/tutorials	2.86	2.65	2.27	1,2,3
35	I find the experience disappointing	2.30	2.35	2.14	**NS**
36	I am able to concentrate well	2.19	2.34	2.09	2
42	The enjoyment outweighs the stress of studying medicine	2.18	2.13	1.76	2,3
43	The atmosphere motivates me as a learner	2.32	2.42	2.00	2,3
49	I feel able to ask the questions I want	1.98	2.54	2.40	1,3
**Students’ Social Self-Perception (SSP)**					
3	There is a good support system for students who get stressed	1.74	1.94	1.19	1,2,3
4	I am too tired to enjoy this course	2.00	2.22	1.57	1,2,3
14	I am rarely bored on this course	1.97	1.83	1.28	2,3
15	I have good friends in this school	3.04	3.29	3.32	1,3
19	My social life is good	2.55	2.73	2.15	2,3
28	I seldom feel lonely	2.23	2.27	2.26	**NS**
46	My accommodation is pleasant	2.97	3.10	3.14	**NS**

1 represents p<0.05 difference between year 1 and 3

2 represents p<0.05 difference between year 3 and 5

3 represents p<0.05 difference between year 1 and 5

NS: not significant
